# Fucoxanthin Holds Potential to Become a Drug Adjuvant in Breast Cancer Treatment: Evidence from 2D and 3D Cell Cultures

**DOI:** 10.3390/molecules26144288

**Published:** 2021-07-15

**Authors:** Fernanda Malhão, Ana Catarina Macedo, Carla Costa, Eduardo Rocha, Alice Abreu Ramos

**Affiliations:** 1 Institute of Biomedical Sciences Abel Salazar (ICBAS), University of Porto (U.Porto), Rua de Jorge Viterbo Ferreira 228, 4050-313 Porto, Portugal; fcmalhao@icbas.up.pt (F.M.); acfpmacedo@gmail.com (A.C.M.); ramosalic@gmail.com (A.A.R.); 2 Interdisciplinary Center for Marine and Environmental Research (CIIMAR), University of Porto (U.Porto), Avenida General Norton de Matos, 4450-208 Matosinhos, Portugal; 3 Environmental Health Department, National Health Institute Dr. Ricardo Jorge, Rua Alexandre Herculano 321, 4000-055 Porto, Portugal; carla.trindade@insa.min-saude.pt; 4 EPIUnit—Instituto de Saúde Pública, University of Porto (U.Porto), Rua das Taipas 135, 4050-600 Porto, Portugal

**Keywords:** cisplatin, combinatorial therapy, doxorubicin, fucoxanthin, seaweed compounds, triple-negative breast cancer

## Abstract

Fucoxanthin (Fx) is a carotenoid derived from marine organisms that exhibits anticancer activities. However, its role as a potential drug adjuvant in breast cancer (BC) treatment is still poorly explored. Firstly, this study investigated the cytotoxic effects of Fx alone and combined with doxorubicin (Dox) and cisplatin (Cis) on a panel of 2D-cultured BC cell lines (MCF7, SKBR3 and MDA-MB-231) and one non-tumoral cell line (MCF12A). Fucoxanthin induced cytotoxicity against all the cell lines and potentiated Dox cytotoxic effects towards the SKBR3 and MDA-MB-231 cells. The combination triggering the highest cytotoxicity (Fx 10 µM + Dox 1 µM in MDA-MB-231) additionally showed significant induction of cell death and genotoxic effects, relative to control. In sequence, the same combination was tested on 3D cultures using a multi-endpoint approach involving bioactivity assays and microscopy techniques. Similar to 2D cultures, the combination of Fx and Dox showed higher cytotoxic effects on 3D cultures compared to the isolated compounds. Furthermore, this combination increased the number of apoptotic cells, decreased cell proliferation, and caused structural and ultrastructural damages on the 3D models. Overall, our findings suggest Fx has potential to become an adjuvant for Dox chemotherapy regimens in BC treatment.

## 1. Introduction

Nature has always been a source of active substances for drug development, and despite the advances in synthetic biology, most of the currently approved medicines are based on natural products [1,2]. In fact, more than 60% of the anticancer drugs used in clinical practice are derived from natural sources, including the well-known chemotherapeutics doxorubicin (Dox), paclitaxel, vincristine and vinblastine [1,3]. In the last few decades, there has been a growing interest in exploring the marine ecosystem for drug discoveries [4,5]. Marine organisms yield a wide variety of bioactive compounds with unique properties and promising potential for developing new anticancer drugs [6,7]. Recent data report that, so far, five marine-derived drugs have been approved for cancer treatment [8], while 24 drug candidates are being tested in clinical trials [9].

Among marine organisms, seaweeds (or macroalgae) bear a high content of phytochemicals (e.g., carotenoids, polyphenolic compounds and polysaccharides) with promising chemopreventive and chemotherapeutic properties towards several types of cancer, namely breast cancer (BC) [10]. Interestingly, in Asiatic folk medicine, seaweeds have been used since ancient times as potential weapons to treat BC [11]. Besides, some studies have also been pointing out the benefits of dietary seaweed consumption on the prevention of BC [12,13].

Fucoxanthin (Fx) is one of the most abundant xanthophyll carotenoids of the marine environment that is mainly found in brown seaweeds such as the popular edible algae Wakame (*Undaria* *pinnatifida*) [14,15]. This orange-colored pigment is known for having distinct health-promoting activities, such as anti-inflammatory, antioxidant, antidiabetic, anti-obesity and anticancer effects in various types of cancers [16,17,18,19,20,21,22], including BC [23]. The anticancer effects comprise proapoptotic, antiproliferative, antimetastatic and antiangiogenic activities [24]. In BC cells, Fx increases apoptosis [14,22,23] and decreases lymphangiogenesis [25].

BC is currently the most prevalent cancer and the leading cause of cancer-related deaths amongst females worldwide [26,27]. In 2020, there were 2.3 million women diagnosed with this disease, accounting for 685,000 deaths globally [28]. BC comprises tumors with high heterogeneity and a wide variety of histological and molecular features, which translate into distinct biological behaviors and treatments [29]. According to the St. Gallen International Breast Cancer Conference 2013, BC is classified into four molecular subtypes: Luminal A, Luminal B, HER-2 overexpression, and triple-negative. These BC subtypes are differentiated by the presence of estrogen receptor (ER), progesterone receptor (PgR) and different expression levels of the human epidermal growth factor receptor 2 (HER-2) and antigen Ki67 [30]. Luminal A (ER+/PgR+/HER-2– with low ki67) and B (ER+/PgR+/HER-2– with high ki67) subtypes present the lowest recurrence rates and the best prognosis [30,31], being typically treated with hormonal therapy or a combination of both chemotherapy and hormonal treatments [32]. The HER-2 overexpression subtype (ER–/PR–/HER-2+) is linked with an aggressive phenotype and worse prognosis, however, it is often successfully treated with HER-2 targeted therapies, such as trastuzumab and pertuzumab [33]. The triple-negative BC (TNBC) subtype (ER–/PR–/HER-2–) accounts for the poorest clinical outcomes due to its aggressive metastatic behavior and the lack of targeted therapies, being chemotherapy the single treatment option currently available [34]. Despite being the most chemotherapy-responsive subtype, TNBCs still present the highest recurrence and metastasis rate compared to other BCs [35].

Current neoadjuvant or adjuvant chemotherapy treatments for BC usually involve the administration of reference anticancer drugs such as Dox, cisplatin (Cis), or paclitaxel isolated or in combination regimens [36]. While commonly used, chemotherapy drugs can cause severe adverse effects, as they induce cytotoxicity in non-target cells (non-tumorigenic cells) [37]. For instance, Dox is normally associated with cardiotoxicity, myelosuppression, intestinal epithelium lesions [36], and Cis can induce nephrotoxicity, neurotoxicity and hearing impairments [38]. Additionally, the onset of multidrug resistance is still one of the major hurdles of chemotherapy, being responsible for the death of many cancer patients [37]. Therefore, strategies to overcome chemotherapy-associated limitations are in need, encouraging the search for new anticancer drugs or adjuvant drugs.

In this vein, recent reports have shown that the combination of established anticancer drugs with natural products, such as marine-derived compounds, can potentiate drug efficacy and reduce the administered doses, causing the mitigation of the associated adverse effects and preventing the onset of drug resistance [39,40].

Although data are scarce, a few studies tested the effects of Fx in combination with conventional anticancer drugs such as 5-Fluorouracil [41], Imatinib [42] and Dox [43]. A recent review on the mechanisms of the anticancer effects of Fx and its combination with chemotherapy drugs reported that generally the combinations were more effective than either Fx or the drugs alone [44]. However, more mechanistic studies are required to investigate the interactions of Fx with anticancer drugs and elucidate the processes underlying the combinatorial effects. Knowledge on this subject is of utmost importance not only for oncological medicine, but also for the field of nutritional therapy, as seaweeds can be included in diets or be consumed as dietary supplements [45].

Most of the existing data regarding the anticancer effects of Fx were obtained from in vitro studies using monolayer cultures. Despite providing valuable information in the scope of oncology drug discovery, 2D cell culture models present several limitations in predicting drug activity and toxicity in vivo [46,47]. One of the main disadvantages of these models is the lack of interactions between cell–cell and cell–extracellular environment. These are usually found in solid tumors, affecting cell polarity and other related cellular functions, including proliferation and differentiation, viability, apoptosis, proteins and gene expression, response to stimuli, and drug metabolism [48]. Accumulating evidence confirms that cells grown in a 3D physical shape have a better predictive capacity of in vivo cellular responses than cells grown as a monolayer [49,50].

To make up for the current lack of information, this study aimed to: (1) evaluate the cytotoxic effects of Fx alone and in combination with reference drugs—Cis or Dox—on a panel of four 2D-cultured cell lines (three BC cell lines of different molecular subtypes and one non-tumoral breast cell line); (2) select the combination with the most promising cytotoxic effect and further investigate its effects in the respective 3D cell model; and (3) explore some possible underlying anticancer mechanisms of that combination on both cell models, using bioactivity assays and microscopy techniques.

## 2. Results

### 2.1. Phase 1—Cytotoxic Effects of Fx Alone in 2D Cell Cultures

**Figure 1:**
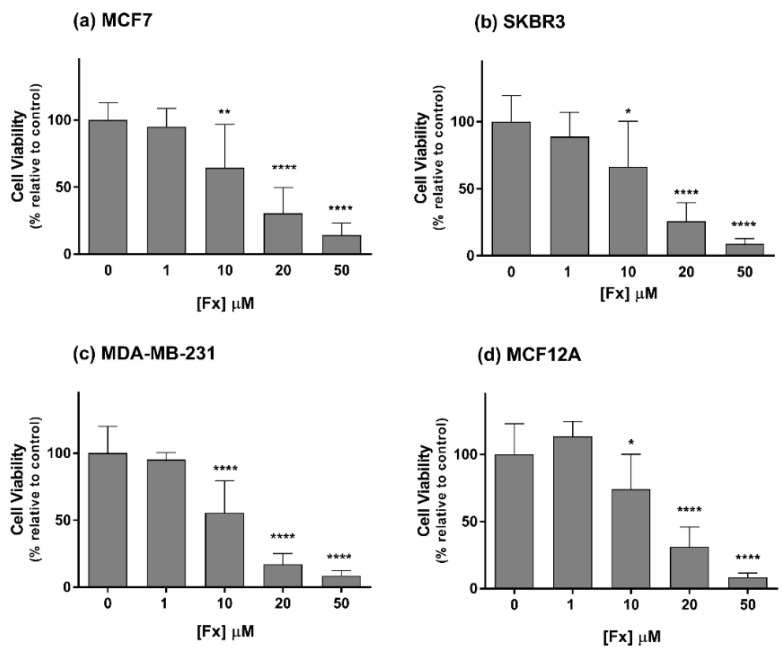
Effects of Fx on the viability of MCF7 (**a**), SKBR3 (**b**), MDA-MB-231 (**c**) and MCF12A (**d**) cells, assessed by the MTT assay after 72 h of incubation. Results are expressed as mean + standard deviation of six independent experiments. Asterisks indicate significant differences relative to control (* *p* < 0.05; ** *p* < 0.01; **** *p* < 0.0001).

The cytotoxicity of Fx was evaluated by the MTT assay after 72 h of exposure. Fucoxanthin induced dose-dependent cytotoxic effects in all the tested cell lines at concentrations equal to or above 10 µM (Figure 1). At 10 and 20 µM, Fx reduced the viability of MCF7, SKBR3 and MDA-MB-231 cells to approximately 62 and 22%, respectively. For the MCF12A cells treated with the same concentrations, the viability decreased to around 74 and 31%. At the highest tested concentration (50 µM), Fx induced significant cytotoxic effects on all the cell lines by reducing their viabilities to nearly 10%.

### 2.2. Phase 2—Cytotoxic Effects of Fx Combined with Dox or Cis in 2D Cell Cultures

Considering the cytotoxicity data and the selection criteria defined in Section 4.4, two concentrations of Fx and two concentrations of Dox and Cis were selected to be tested in combination on the panel of BC cell lines. The effects of these mixtures and respective isolated compounds on cell viability were determined by the MTT assay after 72 h.

**Figure 2:**
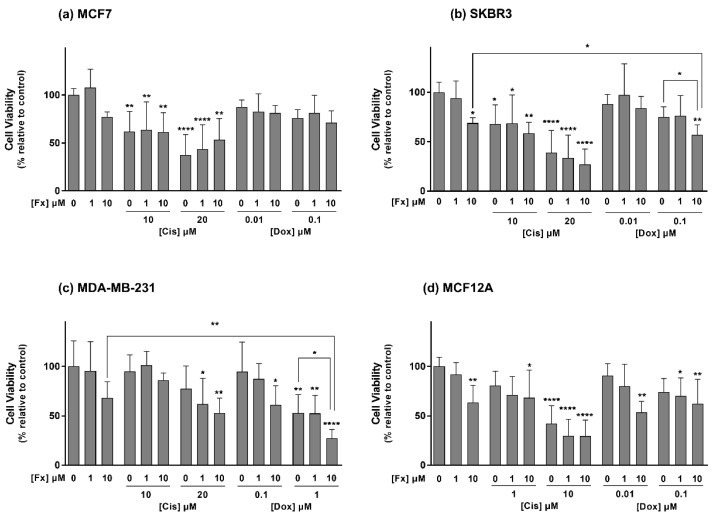
Effects of Fx alone and in combination with Dox or Cis on the viability of MCF7 (**a**), SKBR3 (**b**), MDA-MB-231 (**c**) and MCF12A (**d**) cells, assessed by the MTT assay after 72 h of incubation. Results are expressed as mean + standard deviation of five independent experiments. Asterisks indicate significant differences relative to control (* *p* < 0.05; ** *p* < 0.01; **** *p* < 0.0001). Square brackets indicate differences between conditions using t-tests with sequential Holm–Bonferroni corrections.

Regarding the MCF7 and SKBR3 cells (Figure 2a,b), the drug Cis at 10 and 20 μM markedly reduced the viability of both cell lines by 35% and 62%, respectively. Additionally, all the combinations between this drug and Fx induced significant cytotoxic effects on both cell lines in relation to the control group. Still, those effects did not differ from the ones of the single compounds. Fx at 10 μM induced a significant cytotoxic effect on the SKBR3 cell line, either alone or in combination with Dox at 0.1 μM, resulting in a reduction in cell viability of roughly 31% and 43%, compared to the control. Beyond that, the mentioned combination was also found to cause significantly higher cytotoxicity than the compounds alone, resulting in a cell viability loss of 12% in relation to Fx and 18% compared to Dox.

Results from the MDA-MB-231 cell line (Figure 2c) showed that Cis alone did not induce statistically significant cytotoxic effects. However, the combination of Fx (1 or 10 μM) with Cis 20 μM decreased cell viability in relation to the control. Moreover, cells treated with Dox alone at 1 μM as well as with the combinations Fx 10 μM + Dox 0.1 μM and Fx 1/10 μM + Dox 1 μM also presented a statistically significant decrease in cell viability compared to control cells. Noteworthy, the combination of Dox 1 μM with Fx 10 μM was the only one that remarkably decreased MDA-MB-231 viability to a percentage that statistically differed from both Fx and the drug alone. This combination was able to cause a reduction in cell viability of 73%, in relation to the control, and to increase Dox cytotoxicity by approximately 26%.

In line with the one-way ANOVA analysis detecting differences between groups in the MDA-MB-231 and MCF7 cell lines, the Holm–Bonferroni corrected t-tests comparing only the Fx 10 μM with control (*p* ˂ 0.05 and *p* ˂ 0.001, respectively) indicated that Fx induced statistically significant cytotoxicity in those cells.

Concerning the MCF12A cell line (Figure 2d), Fx at 10 μM exhibited significant cytotoxic effects, reducing cell viability by 37% relative to the control group. Cytotoxicity was also observed in cells exposed to Cis 1 μM combined with Fx 10 μM and to Cis 10 μM, alone and combined with Fx 1 or 10 μM. Furthermore, Dox only significantly increased cytotoxicity in MCF12A cells when combined with Fx 1 μM (Dox 0.1 μM) or Fx 10 μM (Dox 0.01 and 0.1 μM).

Given all the above results, the condition Fx 10 μM + Dox 1 μM tested in MDA-MB-231 cells was elected as the most promising combination since it showed the highest decrease in cell viability, which statistically differed from the control and both the drug and Fx alone. To explore the possible mechanisms of action underlying its cytotoxic activity, the nuclear condensation and comet assays were performed in 2D cultures.

### 2.3. Phase 3—Effects of Fx Combined with Dox on Cell Death and DNA Damage in 2D Cell Cultures

**Figure 3:**
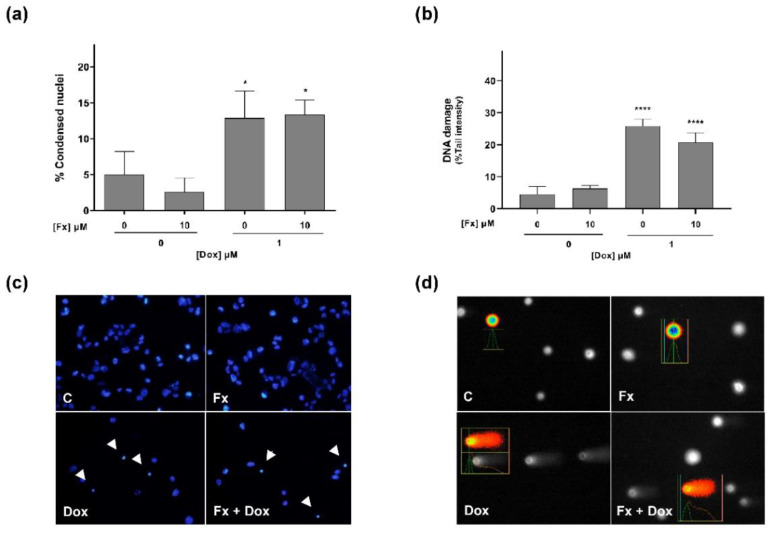
Effects of Fx alone and in combination with Dox on the cell death (**a**,**c**) and DNA damage (**b**,**d**) of the MDA-MB-231 cell line, assessed by the nuclear condensation and comet assays, respectively. Images illustrate the induced cell death (**b**) and genotoxic effects (**d**). The white arrowheads indicate the cells with condensed nuclei. Results are expressed as mean + standard deviation of three to five independent experiments. Asterisks indicate significant differences relative to control (* *p* < 0.05; **** *p* < 0.0001). Scale Bar: 100 µm.

The effect of the selected combination and respective isolated compounds on the induction of cell death in MDA-MB-231 cells was evaluated by the nuclear condensation assay after 72 h of exposure. Statistical results (Figure 3a) showed that Fx alone did not induce effects on cell death. On the other hand, Dox 1 μM and the combination Fx 10 μM + Dox 1 μM markedly increased the number of cells with condensed nuclei (both around 13%), relative to control. Concordantly, when analyzing the images from the nuclear condensation assay (Figure 3c), it was noted that those conditions exhibited a higher number of cells with condensed nuclei, as compared to Fx and the control.

Effects on DNA damage (strand breaks) were determined by the alkaline version of the comet assay following 2 h of exposure. According to the statistical results (Figure 3b), Dox alone and in combination with Fx showed a significant increase in DNA damages compared to the control, accounting for a tail intensity of 26% and 21%, respectively. In both conditions, it was observed an increase of fluorescence intensity in the comet tail (Figure 3d). In contrast, both the control and Fx-treated groups presented a low level of DNA damages, as the DNA of most cells occurred as a nucleoid with no or a small tail.

### 2.4. Phase 4—Effects of Fx Combined with Dox in 3D Cell Cultures (Multicellular Aggregates-MCAs)

#### 2.4.1. Cytotoxic Effects

The most promising combination in the 2D cell culture screening (Fx 10 µM + Dox 1 µM) was selected to be tested in 3D culture. Additionally, combinations with higher concentrations of Fx (20 µM) and Dox (2 µM) were included, as well as a positive control for cytotoxicity (Dox 5 µM). Effects on cell viability were assessed by two assays: MTT and LDH.

**Figure 4:**
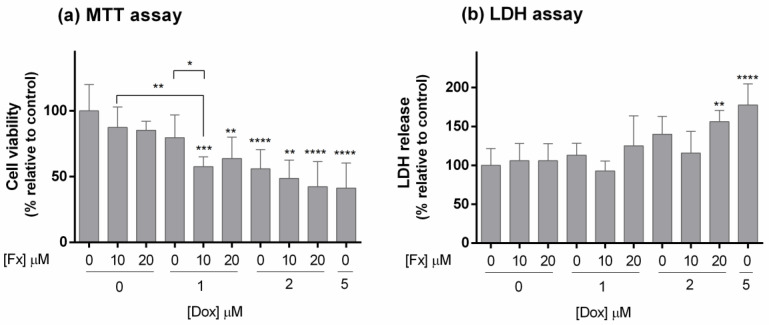
Effects of Fx alone and in combination with Dox on the cell viability of MDA-MB-231 MCAs, assessed by the MTT (**a**) and LDH (**b**) assays after 96 h of incubation. The percentages of cell viability and LDH release are relative to the control and presented as mean + standard deviation of five independent experiments (each in triplicate). Asterisks indicate significant differences relative to control (* *p* < 0.05; ** *p* < 0.01, *** *p* < 0.001; **** *p* < 0.0001). Square brackets indicate differences between conditions using *t*-tests with sequential Holm–Bonferroni corrections.

According to the MTT assay (Figure 4a), Fx alone did not present effects on cell viability in MDA-MB-231 MCAs. Doxorubicin (≥2 µM) and respective combinations showed cytotoxicity, with cell viabilities under 55% in relation to the control. At 1 µM, Dox alone did not significantly affect cell viability, but in combination with Fx 10 µM, it statistically differed from the control and the drug, causing a decrease in 22% of cell viability in relation to the drug and 30% in relation to the seaweed compound alone. As for the other combinations with higher Fx and Dox concentrations, they did not statistically differ from Dox alone.

In the LDH assay (Figure 4b), Fx alone did not present cytotoxic effects on the MDA-MB-231 MCAs. The same was observed after exposure to Dox 1 µM (alone and combined) and Dox 2 µM (alone and combined with Fx 10 µM). The conditions that presented higher LDH release, differing from the control and indicating high cytotoxicity, were Dox 2 µM combined with Fx 20 µM (56%) and Dox 5 µM (77%).

#### 2.4.2. Stereomicroscopic Analysis and Area Measurements

**Figure 5:**
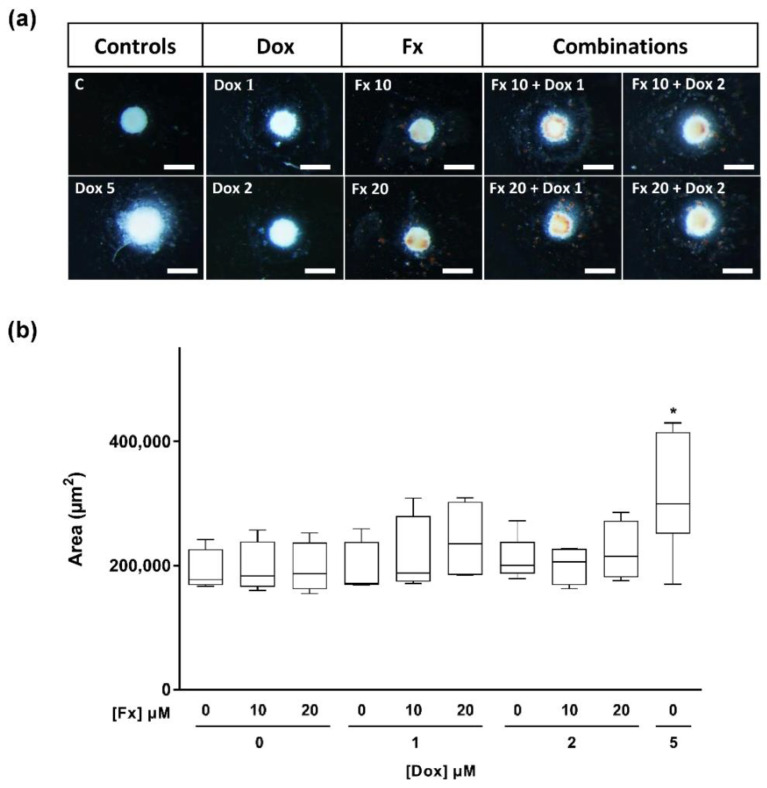
Representative stereomicroscopic images of MDA-MB-231 MCAs exposed to Fx (10 and 20 µM) and Dox (1, 2 and 5 µM), alone and combined, for 96 h (**a**). MCAs area measurements are expressed as median, maximum, minimum and interquartile range (Q3–Q1) of four to six independent experiments (**b**). Asterisks indicate significant differences relative to control (* *p* < 0.05). Scale bar: 500 μm.

MCAs were monitored throughout the exposure by regular observation under a stereomicroscope (Figure 5a), and photographs of each MCA were taken at the end of the exposure period (96 h). To explore the single and combinatory effects of Fx and Dox on the MCAs morphology, the areas of each MCA were measured by the AnaSP software. Results indicated that only the MCAs exposed to Dox 5 μM presented a significant increase in area, compared to control (Figure 5b).

#### 2.4.3. Structural and Ultrastructural Analysis

At the end of the exposure time, MCAs from all tested conditions were fixed, and processed for light and electron microscopy. Paraffin sections were submitted to Hematoxylin-Eosin (HE) staining for assessing the general morphology and for Immunocytochemistry (ICC) analysis to study the apoptotic and proliferative status of the MCAs. Additionally, semithin and ultrathin sections of epoxy-embedded MCAs were obtained to analyze the ultrastructural changes caused by the compounds.

##### HE Staining

**Figure 6:**
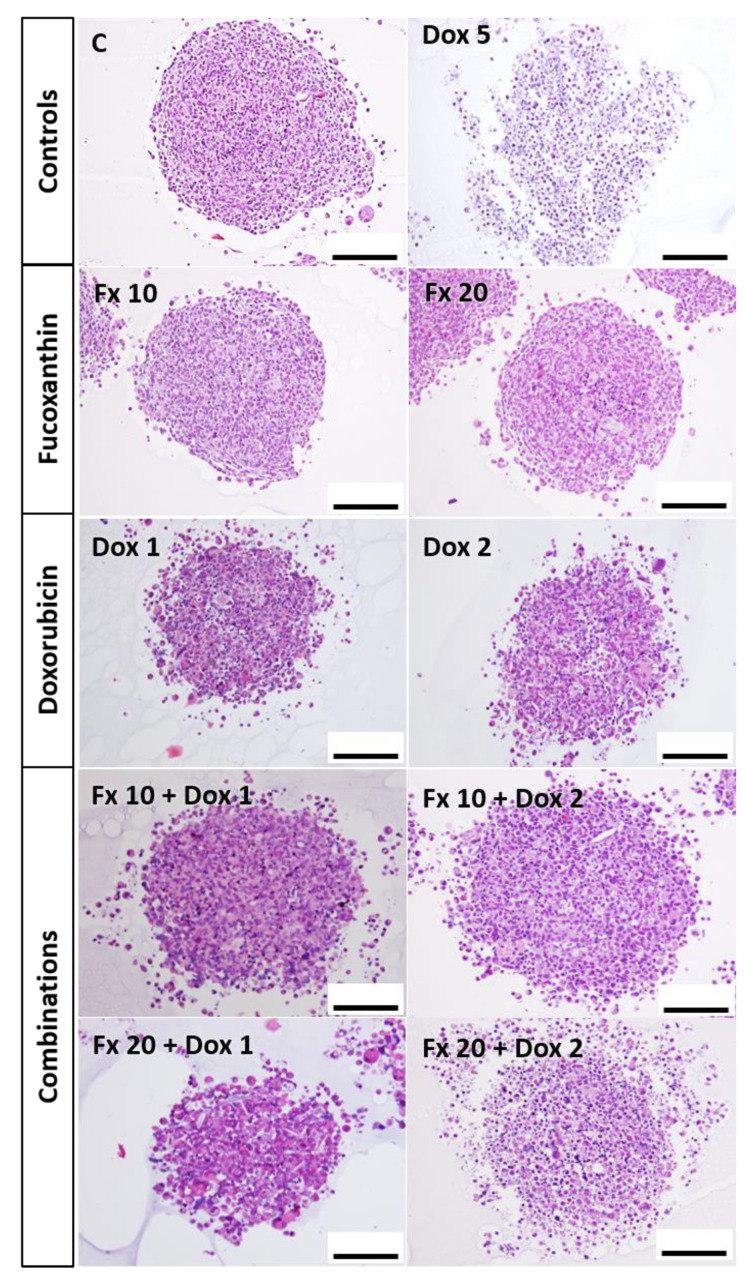
Representative histological images of the MDA-MB-231 MCAs exposed to Fx (10 and 20 µM) and Dox (1 and 2 µM), alone and combined, for 96 h. Dox 5 µM was included as a positive control. MCAs from at least three independent experiments were sectioned and stained with HE staining. Scale bar: 100 µm.

Observation of the HE-stained MCAs (Figure 6) revealed that most MCAs presented a compact structure. This compactness was gradually lost in the exposure conditions of Dox 2 µM > Fx 20 µM + Dox 2 µM > Dox 5 µM. There was a total disaggregation of the MCAs exposed to Dox 5 µM when collecting them for fixation and processing. In all MCAs, there were some cells with hyperchromatic or pyknotic nuclei; however, these features were more evident in Dox 2 µM (alone and combined) and Dox 5 µM.

A higher degradation of the MCAs structure was noticed in the aggregates exposed to the combinations of Fx with Dox, when compared to MCAs treated with the drug alone, especially in the mixtures with Dox plus Fx 20 µM. These MCAs revealed an increased number of cells with an apoptotic morphology comprising pyknotic nuclei, and/or nuclear fragmentation. At the combination of Fx 20 µM with Dox 1 µM, it was observed a marked increase in cellular eosinophilia. The same was not so evident in the combination of Fx 20 µM with Dox 2 µM. No necrotic cores were observed in the sectioned MCAs.

##### ICC

**Figure 7:**
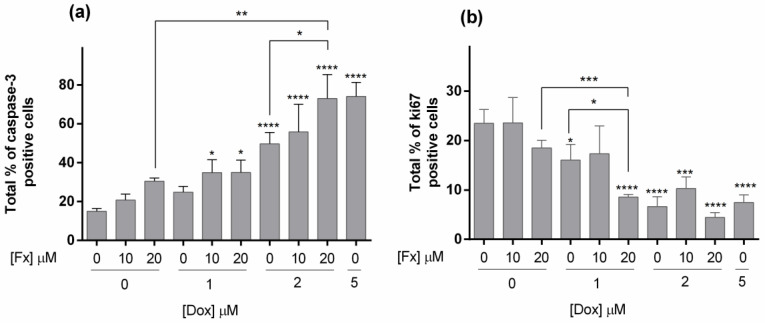
ICC against caspase-3 (**a**) and ki67 (**b**) in MDA-MB-231 MCAs exposed to Fx alone and in combination with Dox for 96 h. Dox 5 µM was included as a positive control. The results are expressed as absolute percentages and presented as mean + standard deviation of three independent experiments. Asterisks indicate significant differences relative to control (* *p* < 0.05; ** *p* < 0.01; *** *p* < 0.001; **** *p* < 0.0001). Square brackets indicate differences between conditions using t-tests with sequential Holm–Bonferroni corrections.

**Figure 8:**
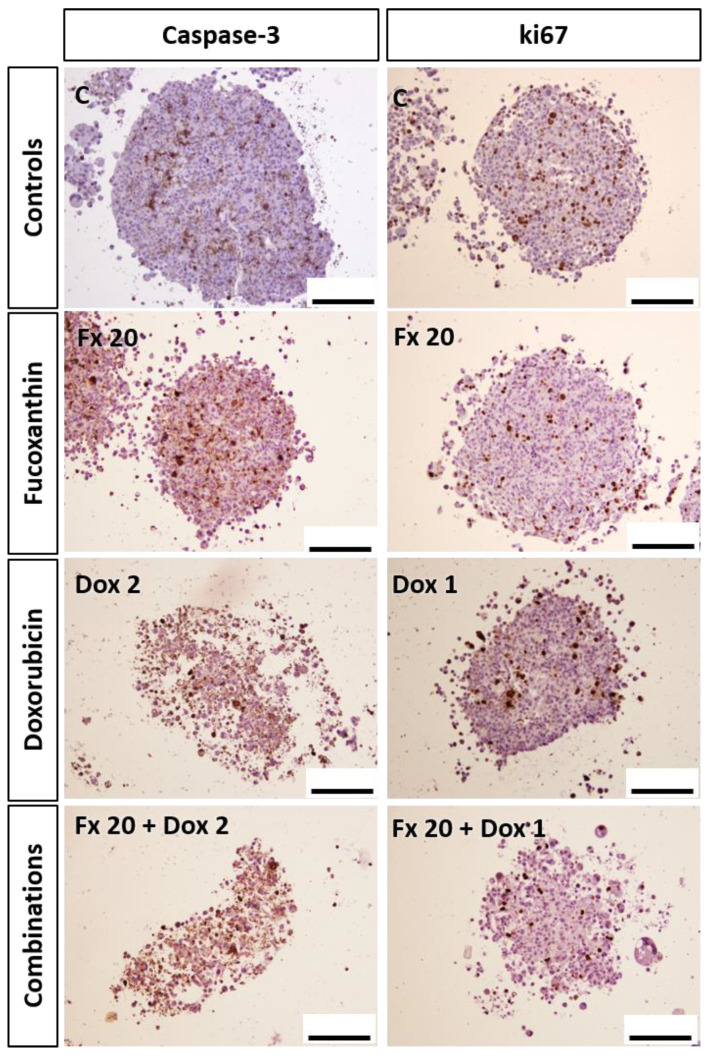
Representative images of ICC against caspase-3 and ki67 in the MDA-MB-231 MCAs exposed to Fx (20 µM) and Dox (1 and 2 µM), alone and combined, for 96 h. Brown staining corresponds to positive immunomarking. Scale bar: 100 µm.

An ICC analysis was performed on the MCAs from all the tested conditions, and the correspondent percentages of positive cells for caspase-3 and ki67 are displayed in Figure 7. About caspase-3 (Figure 7a), the control MCAs presented, on average, 15% of positive cells, revealed by brown staining in the cytoplasm of apoptotic cells (Figure 8). Fucoxanthin and Dox 1 µM alone did not differ from the control. However, when Dox 1 µM is combined with Fx 10 and 20 µM, statistical differences relative to control were found, presenting a significantly higher number of apoptotic cells. Additionally, Dox 2 µM alone and in combinations with Fx, statistically differed from the control. Still, only the combination of Fx 20 µM + Dox 2 µM differed from the control and both compounds alone, presenting an increase of 23% of positive cells relative to Dox 2 µM alone. Figure 8 shows representative images of these conditions immunomarked with caspase-3. Most cells of the MCAs exposed to Fx 20 µM + Dox 2 µM are brown stained, contrasting with the control where the brown staining is restricted to a small number of cells distributed throughout the MCAs. The conditions Fx 20 µM + Dox 2 µM and Dox 5 µM showed marked apoptotic effects as the percentages of positive cells for caspase-3 were five times higher than the control.

Regarding the ki67 proliferation marker (Figure 7b), there was an opposite trend as the number of positive cells decreased with the compound’s concentration increase. The control MCAs showed, on average, 23% of immunomarked cells, corresponding to the brown color in the nucleus (Figure 8). The ki67 positive cells were distributed in the MCAs without any preferential localization. Fx alone did not differ from the control, however, the combination of Fx 20 µM with Dox 1 µM showed a decrease in the number of proliferating cells (8.5% on average) that not only differed from the control but also from both Fx 20 µM (18%) and Dox 1 µM (16%) alone. Representative images of ki67 immunostaining relative to this combination are given in Figure 8. Dox 2 µM (alone and combined) and Dox 5 µM differed from the control, showing fewer ki67-positive cells.

##### Transmission Electron Microscopy (TEM)

**Figure 9:**
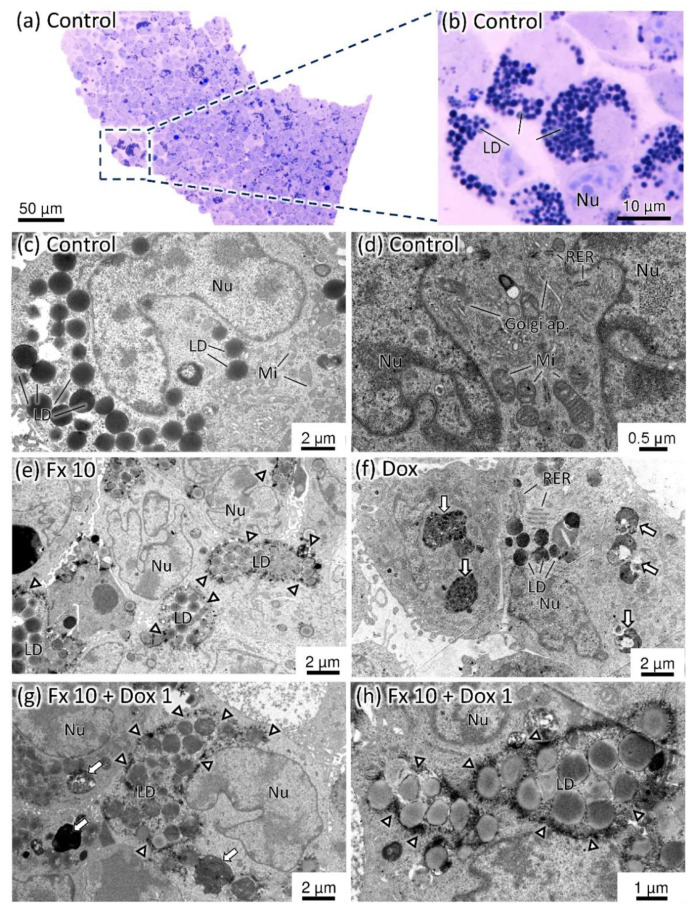
Representative images of semithin (**a**,**b**) and ultrathin sections (**c**–**h**) of MDA-MB-231 MCAs after 96 h of incubation. Golgi ap.—Golgi apparatus; LD—lipid droplets; Mi—mitochondria; Nu—nucleus; RER—rough endoplasmic reticulum. The arrows indicate the autophagic vacuoles and arrowheads point to the electron-dense granular deposits around lipid droplets.

The observation of the MCAs semithin sections revealed a subset of cells with high cytoplasmic lipid content. These cells were present in all tested conditions, even in the control group (Figure 9a,b). The study of the MCAs ultrathin sections confirmed a high amount of lipid droplets in some cells (Figure 9c). Generally, cells possessed very irregular nuclei, a cytoplasm rich in organelles, constituted mainly by Golgi apparatus, rough endoplasmic reticulum cisternae and irregularly shaped mitochondria (Figure 9c,d).

Figure 9e–h illustrate the ultrastructural changes found in the most promising combination (Fx 10 µM + Dox 1 µM).

The MCAs exposed to Dox alone and Dox combined exhibited cells with dense bodies and pleomorphic autophagic vacuoles (Figure 9f–h), which were more pronounced at the tested conditions that contained Dox 2 µM and Dox 5 µM.

The MCAs exposed to Fx displayed lipid droplets too, but with a lower electron density when compared to those of the control and Dox alone. In these latter conditions, lipid droplets appeared as denser structures. Additionally, in the Fx exposed cells, there was a deposition of fine granular electron-dense material around the lipid droplets (arrowheads in Figure 9e,g,h). These deposits existed in all tested conditions that involved Fx, being more evident at Fx 20 µM.

The MCAs treated with the combinations of Fx and Dox presented the same alterations observed in the MCAS exposed to each compound alone.

## 3. Discussion

Fucoxanthin has been considered as a promising anticancer compound. However, most of the available data regarding its effects derive from in vitro studies performed in 2D cultures, and its underlying mechanisms of action are not fully elucidated. Apart from this, there are just a few studies about its combinatory effects with reference anticancer drugs, especially in BC. Thus, our study aimed to bring new insights into the effects of Fx alone and in combination with two reference drugs (Dox and Cis) in a panel of 2D-cultured breast cell lines and investigate the most promising combination (Fx + drug) in a more physiologically relevant in vitro model—the 3D cell culture. This study is the first to report the effects of Fx in combination with a chemotherapy drug in a BC 3D model.

Considering 2D cultures, Fx exerted cytotoxicity in all the cell lines (tumoral and non-tumoral) in a dose-dependent manner. Even though we found no studies in the literature for the SKBR3 BC cell line, several authors have reported the cytotoxic activities of Fx towards the MCF7 and MDA-MB-231 cells [14,23,51]. For instance, Rwigemera and colleagues stated that Fx promotes a dose-dependent decrease in the metabolic activity of MCF7 and MDA-MB-231 cells, at similar concentrations to the ones used herein [14]. The cytotoxic effects of Fx in the non-tumoral cells are controversial in the literature, since some authors claim the absence of Fx-induced effects on normal cells [21,52], while others as de La Mare and coworkers, showed that Fx at 10 μM reduced the percentage survival of MCF12A cells to around 71% [53]. Our results are in line with the latter one indicating that Fx is not specific to tumor cells since MCF12A cells were also affected.

According to our results, Fx did not induce cell death nor DNA damage detectable by the conducted assays. Nonetheless, some studies suggested that Fx caused apoptotic and genotoxic effects in BC cells [14,23,51]. When analyzing the existing reports, we noted that apart from the differences in the BC cell lines tested, some used different culture conditions, higher concentrations of Fx, different exposure times, and/or distinct experimental methods for analyzing cell death and DNA damage.Besides, the used alkaline version of the comet assay only detects strand-breaks and alkali-labile sites [54], not excluding that other DNA damages might occur. All these factors might explain the divergent outcomes compared to our study.

Regarding the individual effects of the drugs in 2D cultures, Cis reduced the viability of all cell lines except the MDA-MB-231 cells. These results suggest that MDA-MB-231 is more resistant to Cis than other BC cell lines, as Leon-Galicia and colleagues reported [55]. Differently, Dox only showed cytotoxicity in MDA-MB-231 cells at the highest tested concentration. In agreement, other authors also reported cytotoxicity in the MDA-MB-231 cell line at the same Dox concentration [56]. Dox cytotoxicity seems to be correlated to the increase of DNA damages and consequently induction of cell death in MDA-MB-231 cells, concordantly with other reports with the same cell line [57,58]. Dox is known for causing DNA damage through several different mechanisms (e.g., DNA intercalation, topoisomerase II inhibition, ROS induction), which can trigger cell cycle arrest, impairment of mitochondrial function and cell death [59,60].

Drug combinations bring several benefits in cancer treatment as they might, for instance, increase drug efficacy, decrease drug resistance and reduce the adverse effects. In this scenario, the combination of anticancer drugs with natural compounds has been reported in some clinical trials as a promising chemotherapeutic strategy [61]. In this study, the drug concentrations used in the combinatorial experiments were clinically relevant as they were similar to plasmatic concentrations found in oncological patients after intravenous infusion [62]. Besides, they were in line with other studies that used BC lines [63,64].

Our results showed that the combination of Fx with Dox promotes greater cytotoxic effects than each of the compounds separately on the SKBR3 and MDA-MB-231 cell lines. Interestingly, the most promising anticancer effects were noted for the combination Fx 10 μM + Dox 1 μM in the MDA-MB-231 cell line, representative of TNBC, the BC subtype where chemotherapy with drugs like Dox is the unique treatment option.

Recent reports have assessed the effects of Fx combined with conventional drugs in several types of cancer (e.g., leukaemia and colon cancer) [42,44,65]. However, data is almost non-existent in the context of BC, being, as far as we know, restricted to one study performed by Vijay and colleagues [43]. The latter supports our results, showing that the combination of Fx with Dox was more cytotoxic to MDA-MB-231 cells than the individual compounds. Also, in our study, the combination of Dox with Fx promoted genotoxicity and cell death in MDA-MB-231 cells, however, the effects were not different from Dox alone. These findings do not fully explain the increased cytotoxicity registered for this combination. One possible explanation for the observed effect might be the induction of cell cycle arrest since it is one of the mechanisms induced by Fx in different cancer cell lines [66,67]. Some reports also describe that the combination of carotenoids with ROS-inducing anticancer drugs like Dox, can act synergistically, enhancing the toxicity of the drug [68] which probably occur in this case, as Fx can also have a pro-oxidant action, increasing ROS, and consequently triggering cell death pathways [17,69].

Regardless of the inconclusive mechanistic data reported in 2D, the most promising combination was explored in a more complex cell model—3D MCAs. Although many studies reported the effects of Fx in 2D cultures, in 3D cultures, as far as we investigated, the literature is limited to four studies [53,70,71,72]. Only de la Mare and collaborators reported Fx effects in a BC cell line cultured in 3D [53]. However, they aimed to test the impact of Fx on the formation of mammospheres and not in already formed 3D BC cultures. The scarce data on 3D cultures reinforces the need for more studies exploring the effects of Fx alone and combined with the chemotherapy drugs.

It seems now consensual that 3D cell cultures are more resistant to drug treatments and better translate organism-level realities [73,74]. That is why we prolonged the exposure time in MDA-MB-231 3D cultures and tested not only the most promising combination in 2D, but also higher Fx and Dox concentrations alone and in combination.

In the MTT assay, effects on cell viability were observed at Dox 1 µM only when combined with Fx, and at higher concentrations of Dox, alone and combined. Otherwise, in the LDH assay, cytotoxic effects were only observed in two conditions: Fx 20 µM + Dox 2 µM and Dox 5 µM. The used viability tests (MTT and LDH) are both colorimetric assays, although they are based on two different approaches. The MTT assay relies on mitochondrial metabolic activity [75], while the LDH test evaluates plasmatic membrane integrity through the quantification of the LDH released from damaged cells [76]. MTT assay detected differences in cell viability at lower Dox and Fx concentrations, while in LDH, only higher concentrations differed from the control. Thus, the differences in the detected cytotoxicity can be related to the different targets of these assays. The cytotoxic effect of the combination Fx with Dox obtained by the MTT, in 3D cultures, lined up with the results from 2D cultures.

A previous study from our group showed similar results to Dox’s cytotoxicity in MDA-MB-231 cells [77]. Other authors tested Dox’s concentrations that were from 10 [53] to 100 [78] times higher than the ones applied in our study, and surprisingly did not induce greater cytotoxicity.

Furthermore, the morphological evaluation by stereomicroscopy only revealed a significant increase in the area of the MCAs treated with the highest cytotoxic condition. Thus, no significant variations were detected for the conditions with a lower cytotoxicity degree, showing that the evaluation of the MCAs areas alone has not enough sensibility to detect cytotoxic effects.

The histological analysis of the HE-stained MCAs supports the results of the MTT assay. It was observed a deterioration in cell morphology of the MCAs treated with the conditions that statistically differed from the control in MTT. Additionally, the MCAs that revealed the highest degree of morphological damage presented cytotoxic effects that statistically differed from the control in both cytotoxic assays.

Apoptosis is widely used to evaluate the cellular response to a chemotherapeutic agent [79], and caspase-3 is frequently employed as a biomarker of apoptosis [80]. The ICC evaluation of caspase-3 expression showed that Fx alone did not differ from the control, similarly to 2D results from the nuclear condensation assay. The combination of Fx with Dox increased the percentage of caspase-3 positive cells in relation to the drug alone, suggesting a possible pro-apoptotic effect of Fx. Previous studies also reported that Fx induces apoptosis in several cell lines [18,23,61], even in 3D models [72]. However, due to the non-existing data on the combined effects of Fx and Dox in 3D BC cultures, it was not possible to compare our results in such situations.

Additionally, the results of ICC for caspase-3 and MTT in 3D were very similar, as both experiments showed that Fx and Dox 1 µM alone did not present differences in relation to the control, while Dox ≥ 1 µM in combination with Fx revealed differences that show cell cytotoxicity and apoptosis induction. Once again, these findings reinforce the promising effects of combining Fx with Dox.

The effects on cell proliferation were evaluated using ki67, a well-known prognostic marker for BC [81], that is expressed in all phases of the cell cycle, except the G0 phase [82]. In 3D culture, Fx alone did not show effects on cellular proliferation, however, the combination of Fx 20 µM with Dox 1 µM augmented the drug effects, lowering cell proliferation to nearly half of the percentage of the compounds alone. These results point to a possible decrease in the cell proliferation caused by the combination of Fx and Dox. Still, further evaluation of the cell cycle will be necessary to elucidate this topic.

The histological analysis complemented with ICC unveiled as a useful tool for evaluating the cytotoxicity of the tested compounds, not only for corroboration of the cytotoxic assays and verifying the proliferation and apoptotic status, but also to give a general view of the morphology of the MCAs, verify the existence of necrotic cores, and correlate the ICC markers with their localization into the MCAs. An additional advantage of processing MCAs for paraffin embedding is the possibility of having the biological samples in a form that can be stored indefinitely and generate sections for different ICC markers or even extract genetic material for further studies.

The ultrastructural analysis revealed that MDA-MB-231 MCAs presented a pool of cells with high lipid droplets content, not noticeable in paraffin sections due to lipid dissolution during the processing. These lipid droplets were previously reported in this cell line, and the authors described that the sub-population of highly enriched lipid cells was related to stemness features [83]. In MCAs exposed to Fx (alone and combined), a lower electron density of the lipid droplets was observed. Knowing that the electron density of lipid droplets reflects fatty acid composition [84], this can indicate that Fx can influence the composition of the lipid droplets. Additionally, it was noted a deposition of a granular electron-dense material around the lipid droplets. The explanation of such changes needs to be investigated, however, it could be related to lipidic trafficking as Fx is described as a regulator of pathways related to fatty acid synthesis, lipolysis, and thermogenesis [85]. Indeed, the presence of lipid droplets is thought to be part of stress response to treatments, regulation of proliferation, migration and survival of cancer cells [86]. The additional presence of a great number of autophagic vacuoles in the cytoplasm indicates that Dox induced autophagy in the MDA-MB-231 cell line, which agrees with a previous report [87].

In summary, our results indicated that Fx alone had cytotoxic effects in all the 2D-cultured breast cell lines. In combination with Dox, Fx suggestively potentiated the drug effect in SKBR3 and MDA-MB-231 cells, being these effects more pronounced in the TNBC cell line. However, the mechanisms behind the enhanced cytotoxicity need further elucidation, since in addition to DNA damage and cell death induction, other mechanisms as cell cycle arrest, ROS induction and alteration of lipid metabolism may occur.

Besides being more resistant to Fx and Dox alone, 3D cultures also presented higher cytotoxic effects in the combination of Dox with Fx, corroborating the 2D results. The cytotoxicity in 3D was supported by the morphological analysis (light and electron microscopy) and ICC. Apart from increasing the number of apoptotic cells and lowering cell proliferation, the combination of Fx and Dox damaged the MCAs histological structure and caused ultrastructural alterations. These findings reinforce the utility of using a multi-endpoint approach for evaluating the cytotoxic effects of compounds.

Our data from 2D and 3D cultures suggest that Fx has potential as a drug adjuvant inTNBC treatment when Dox is applied for chemotherapy. Notwithstanding, more in vitro and in vivo studies are necessary to explore the underlying mechanisms of action of Fx and its combination with Dox.

## 4. Materials and Methods

### 4.1. Chemicals

3-(4,5-Dimethyl-2-thiazolyl)-2,5-diphenyl-2H-tetrazolium bromide (MTT), 4′,6-diamidino-2-phenylindole (DAPI), cisplatin, cholera toxin, doxorubicin, epidermal growth factor receptor (EGFR), fucoxanthin, hydrocortisone, insulin, low and normal melting agarose and triton X-100 were obtained from Sigma Aldrich (St. Louis, MO, USA). Dimethyl sulfoxide (DMSO) was purchased from VWR Chemicals (Solon, OH, USA). Dulbecco’s modified Eagle’s medium (DMEM) high glucose without glutamine and phenol red, fetal bovine serum (FBS), streptomycin-penicillin and trypsin-ethylenediaminetetraacetic acid (EDTA) solution were acquired from Biochrom KG (Berlin, Germany). Dulbecco’s modified Eagle’s medium/Ham’s nutrient mixture F12 (DMEM/F12) medium without phenol red was obtained from GE Healthcare (Chicago, IL, USA). All the other reagents and chemicals used were analytical grade.

### 4.2. Stock and Exposure Solutions

Stock solutions of Dox and Fx were prepared in DMSO and the stock solution of Cis was prepared in 0.9% NaCl solution. All stock solutions were kept at –20 °C before use, except Cis stock solution that was kept at 4 °C for up to 1 month. Exposure solutions were always prepared before experiments by diluting the appropriate volume of each compound stock solution into the respective supplemented fresh culture medium (DMEM or DMEM/F12 medium, dependent on the cell line).

### 4.3. Cell Culture

#### 4.3.1. 2D Cell Culture

The MDA-MB-231, SKBR3 and MCF12A cell lines were purchased from the American Tissue Culture Collection (ATCC) and the MCF7 cell line was acquired from the European Collection of Authenticated Cell Cultures (ECACC). MCF12A is a non-tumor breast cell line, while the others are tumor cell lines representative of different BC subtypes: MCF7—Luminal A; SKBR3—HER-2 subtype; MDA-MB-231—TNBC [88,89]. MCF7, SKBR3 and MDA-MB-231 cells were cultured in high glucose DMEM deprived of phenol red and supplemented with 10% of FBS and 1% of the streptomycin-penicillin solution. MCF12A cells were cultured in DMEM/F12 supplemented with 20 ng/mL of EGFR, 100 ng/mL of cholera toxin, 0.01 mg/mL of human insulin and 500 ng/mL hydrocortisone, 10% FBS and 1% of streptomycin-penicillin solution. All the cell lines were maintained as monolayer cultures in T75 cm^2^ culture flasks (Orange Scientific, Belgium) and incubated under standard cell culture conditions (37 °C, 5% CO_2_). When reaching approximately 80% confluence, cells were subcultured using 0.25% trypsin/EDTA at 37 °C, counted in a hemocytometer and assessed for their viability using the standard trypan blue staining procedure. All experiments were conducted with cells at passages under 40.

#### 4.3.2. 3D Cell Culture—Multicellular Aggregates (MCAs)

MCAs were formed in ultra-low attachment plates (Corning Inc., Corning, NY, USA) as described in a previous report [90]. Cells were seeded at 40 × 10^4^ cells/mL, 200 µL per well, and MCAs were formed after 72 h of incubation at 37 °C, 5% CO_2_.

### 4.4. Study Design

**Figure 10:**
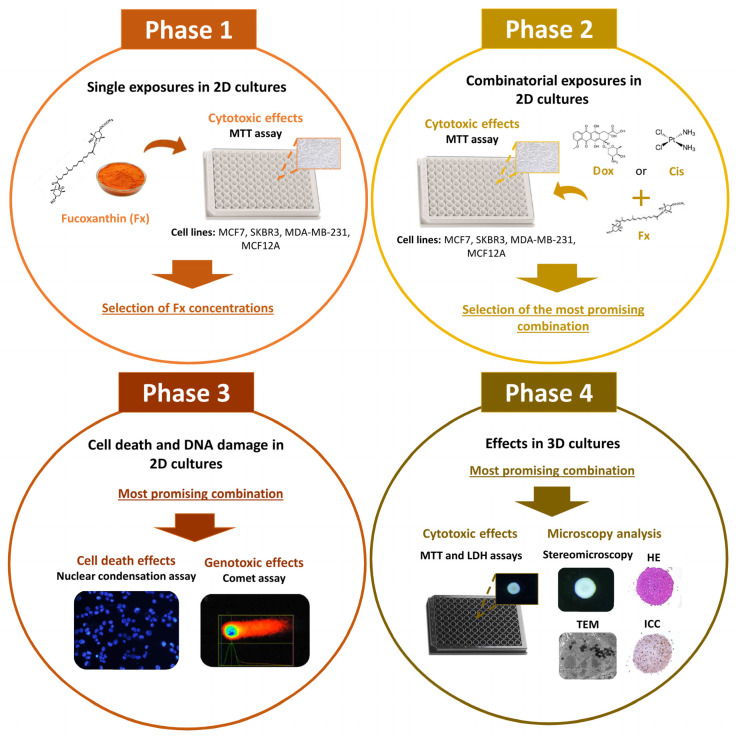
Schematic representation of the study design. HE: hematoxylin and eosin; ICC: immunohistochemistry and TEM: transmission electron microscopy.

Experiments were conducted according to the study design represented in Figure 10. First, in Phase 1, the cytotoxic effects of four concentrations of Fx (1; 10; 20 and 50 µM) were screened by the MTT assay in the panel of breast cell lines cultured in 2D. Considering the results obtained in Phase 1, two concentrations of Fx were selected to be tested in combination with two reference drugs (Cis and Dox) in Phase 2 (Table 1). For each cell line, one concentration of Fx was selected with no statistical effect on cell viability and another that did not reduce the cell viability below 50% (on average). The drug concentrations were chosen according to the results of a recent report [77]. Only the concentrations with no effects or that did not affect cell viability by more than 50% were selected for our study. As in Phase 1, in Phase 2 the cytotoxic effects of the combinations and respective isolated compounds were also assessed by the MTT assay on the 2D-cultured breast cell lines. According to the results obtained in Phase 2, we selected the most promising combination, that is, the one with the highest cytotoxic effects that statistically differed from the control and both isolated compounds. This combination and constituent compounds were further explored in the respective cell line, during Phases 3 and 4. In Phase 3, the nuclear condensation and comet assays were conducted on 2D cultures to study the potential mechanisms behind the induced cytotoxicity. In Phase 4, the combination and respective individual compounds were tested on 3D cultures at equal and higher concentrations than the ones tested in the 2D cultures. The effects were evaluated using a combination of functional and morphological methodologies, including cytotoxic assays (MTT and LDH assays), stereomicroscopy, hematoxylin and eosin (HE) staining, immunocytochemistry (ICC) and transmission electron microscopy (TEM).

In all the experiments, control cells (negative control) were incubated in a culture medium with 0.1% DMSO.

### 4.5. Exposures (Single or Combination) in 2D Cell Cultures

#### 4.5.1. MTT Assay

Cells were seeded in 96-multiwell culture plates (Orange Scientific, Braine-l’Alleud, Belgium) at a density of 0.5 × 10^5^ cells/mL (100 μL/well) and left to adhere for 24 h. Then, cells were exposed for 72 h to different concentrations of Fx alone (Phase 1) and in combination with Dox or Cis (Phase 2). After the exposure, 10 μL (0.5 mg/mL) of MTT stock reagent was added to the wells, and the microplate was incubated for 2 h at 37 °C, 5% CO_2_. The medium was then removed, and the formazan crystals were dissolved in 150 μL of DMSO under slight agitation for 30 min in the dark. Absorbance (A) was measured at 570 nm using a microplate reader Multiskan GO (Thermo Fisher Scientific, Waltman, MA, USA). Results were expressed as % of cell viability relative to the control—an average of five to six independent experiments performed in triplicate—and calculated according to the following equation:

Cell viability (%) = (A_Sample_ ÷ A_Control_) × 100

#### 4.5.2. Nuclear Condensation Assay

Cells were seeded (1000 μL/well) in 24-multiwell culture plates (Orange Scientific, Belgium) at a density of 0.5 × 10^5^ cells/mL and incubated for 24 h under standard cell culture conditions. Cells were then exposed for 72 h to the most promising combination selected in Phase 2 and respective isolated compounds. Briefly, both adherent and non-adherent cells were collected, washed, centrifuged and fixated with 4% (*w*/*v*) paraformaldehyde (Sigma Aldrich, St. Louis, MO, USA) in PBS. Then, cells were placed onto silane adhesive microscope slides (VWR International B.V, Amsterdam, The Netherlands) by cytocentrifugation using Shandon Cytospin 3 cytocentrifuge (Thermo Fisher Scientific, Waltham, MA, USA) at 28× *g* for 5 min. Slides were incubated with DAPI staining solution (1 μg/mL) for 10 min in the dark and after incubation, at least 300 cells per sample were counted under a fluorescence microscope (Olympus IX71, Tokyo, Japan) using the total magnification of 200× (20× objective lens plus 10× eyepiece lens). The results were expressed as percentage of condensed nuclei—an average of three to five independent experiments, with one replicate per condition—and calculated according to the following equation:

% Condensed nuclei = (No. _cells with nuclear condensation_ ÷ No. _total cells_) × 100

#### 4.5.3. Comet Assay

Cells were seeded at 0.5 × 10^5^ cells/mL (1000 μL/well) in 24-multiwell culture plates and allowed to adhere for 24 h, under standard conditions. Subsequently, cells were treated for 2 h with the test conditions and by the end of the treatments they were washed with PBS, trypsinized with 0.25% Trypsin/EDTA solution, collected and centrifuged at 1700× *g* for 1 min using the Micro Star 12 microcentrifuge (VWR International, Pennsylvania, USA). After centrifugation and removal of the supernatant, the cells were mixed with 0.5% (*w*/*v*) Low Melting Point Agarose and transferred to microscope slides previously coated with 1% (*w*/*v*) Normal Melting Point Agarose. Slides were immediately covered with glass coverslips (Thermo Fisher Scientific, Waltham, MA, USA) and maintained at 4 °C for 10 min. Then, the coverslips were removed, and slides were incubated in a lysis solution [2.5 M NaCl, 100 mM Na_2_EDTA, 10 mM Tris Base, pH 10 plus 1% (*v*/*v*) Triton X-100, pH 10] for 1 h, at 4 °C, to lysate the cells and release the DNA. Subsequently, slides were washed with distilled water, transferred into a horizontal electrophoresis tank, and immersed in electrophoresis buffer (300 mM NaOH and 1 mM Na_2_EDTA, pH ≥ 13) for 40 min at 4 °C for DNA unwinding. Electrophoresis ran for 20 min at 4 °C under a voltage gradient of 1 V per cm (20 V). Following electrophoresis, slides were rinsed in distilled water, dehydrated with absolute ethanol, and lastly air-dried. Before analysis, slides were rehydrated in 25 mL of Tris-EDTA (TE) Buffer (Tris-HCl 10 mM and EDTA 1 mM) for 15 min under slight agitation, and then 20 μL of SYBR Gold (Thermo Fisher Scientific, Waltman, MA, USA) was added to the TE buffer for DNA staining. Slides were incubated for 30 min in the dark under slight agitation. After staining, slides were analyzed with a Nikon Eclipse E400 microscope (Nikon, Tokyo, Japan) connected to an epi-fluorescence illuminator Nikon C-SHG1 power supply for HG 100 W with 250× magnification (Semrock SYBRGold-A-NQF filter, Rochester, NY, USA). Samples were analyzed by the Comet Assay IV^TM^ software (Perceptive Instruments, Haverhill, UK) and the parameter “tail intensity” (percentage of DNA in the comet tail) was used to evaluate the DNA damages. A hundred randomly selected nucleoids were analyzed per sample. Results were expressed as the mean of three independent experiments, with one replicate per condition.

### 4.6. Exposures (Single or Combination) in 3D Cell Cultures

MCAs were exposed to Fx alone and combined with Dox for 96 h under standard cell culture conditions. Cells treated with 0.1% DMSO and Dox 5 µM were included as a negative and positive control, respectively.

#### 4.6.1. MTT Assay

The MTT assay was performed as previously described for monolayer cultures, with slight modifications. MCAs were incubated in the dark at 37 °C in a 5% CO_2_ atmosphere with 20 μL of the MTT stock solution per well for 4 h. After incubation, the MCAs were transferred from the ULA 96-well microplates into 96-well flat-bottomed microplates and the medium was removed. Formazan crystals were dissolved in 150 μL of DMSO and after 30 min under slight agitation in the dark, absorbance measurements were performed. The cell viability percentages were calculated according to the formula mentioned above. The results were expressed as the mean of five independent experiments performed in triplicate.

#### 4.6.2. Lactate Dehydrogenase (LDH) Assay

LDH assay was performed using the LDH Cytotoxicity Assay Kit (Enzo Life Sciences, Lausen, Switzerland) according to the manufacturer’s instructions. The medium from the treated conditions, high control (maximum LDH release by applying the lysis buffer) and negative control wells were transferred to a flat-bottom 96-well microplate. Then, the working solution from the LDH kit was added and after the incubation period, the reaction was terminated by the stop solution. Absorbance (A) was measured at 490 nm in a microplate reader Multiskan GO (Thermo Fisher Scientific, Waltman, MA, USA). The results were expressed as percentage of cytotoxicity based on the LDH release—mean of five independent experiments performed in triplicate—and calculated using the following equation:

Cytotoxicity (%) = (A_test substance_ − A_negative control_) ÷ (A_high control_ − A_negative control_) ×100

#### 4.6.3. Stereomicroscopic Analysis and Area Measurements

MCAs were photographed at the end of the exposure (96 h) using an Olympus SZX10 stereomicroscope, equipped with a digital camera DP21 (Olympus, Tokyo, Japan). Stereomicroscope images were analyzed by the free download AnaSP software [91] that measured the MCAs areas. Results were expressed as the mean of four independent experiments performed in triplicate.

#### 4.6.4. Hematoxylin and Eosin (HE) Staining

MCAs were fixed, harvested, processed, embedded in paraffin blocks and ultimately sectioned as previously described [77]. The obtained slides were selected and divided for standard HE staining and immunocytochemistry (ICC). For HE, sections were deparaffinized in xylene (twice, 10 min each), rehydrated in descending alcoholic concentration solutions (absolute ethanol, ethanol 95% and ethanol 70%, 5 min each) and rinsed in running tap water. Then, sections were stained with Mayer’s hematoxylin (Merck, Darmstadt, Germany) for 2 min, rinsed in tap water, stained with eosin Y 1% aqueous solution (Merck, Darmstadt, Germany) for 5 min and newly washed in tap water. Following the HE staining, sections were dehydrated in absolute ethanol (thrice, 5 min each) and diaphanized in xylene (twice, 3 min each). Finally, slides were mounted using Coverquick 2000 mounting medium (VWR International, France). Representative images of MCAs sections were taken using a DP21 camera (Olympus, Tokyo, Japan) linked to an Olympus BX50 microscope (Olympus, Tokyo, Japan).

#### 4.6.5. Immunocytochemical Analysis

ICC was performed using ki67 and caspase-3 as proliferation [92] and apoptosis [93] markers, respectively. Sections of the paraffin-embedded MCAs were deparaffinized and hydrated as described for the HE staining protocol. The heat antigen retrieval step was performed in a pressure cooker by placing the slides in boiling citrate buffer (0.01 M, pH 6.0) for 2 min after reaching the maximum pressure. After cooling and rinsing with distilled water, slides were immersed for 10 min in a solution of 3% hydrogen peroxide in methanol to block endogenous peroxidase activity and rinsed in tris-buffered saline (TBS, pH 7.6). Briefly, unspecific antibody binding was blocked for 5 min using the Protein block reagent from the NovoLinkTM Max Polymer Detection System Kit (Leica Biosystems, Nussloch, Germany) and the sections were rinsed with TBS (twice, 5 min each). Thereafter, the sections were incubated for 2 h in a humidified chamber at room temperature with the following primary antibodies: Rabbit monoclonal anti-Ki67, clone SP6 (Biocare Medical, Pacheco, CA, USA), dilution of 1:200; rabbit polyclonal anti-caspase-3, ab 13847 (Abcam, Cambridge, UK), dilution 1:5000. All the primary antibodies were diluted in PBS with 5% bovine serum albumin (BSA) (Nzytech, Lisbon, Portugal). As a negative control, sections were incubated in PBS with 5% BSA solution under the same conditions. After incubation, sections were rinsed (twice, 5 min each) in TBST (TBS with 0.05% Tween 20) (Sigma Aldrich, St.Louis, MO, USA), incubated for 30 min with the Post Primary solution from the kit and again washed with TBST. Slides were incubated with the NovoLinkTM Polymer reagent (30 min), washed in TBST (twice, 5 min each) incubated with DAB (3,3′-diaminobenzidine) working solution from the kit for signal revelation (2 min) and rinsed in tap water. Lastly, slides were counterstained with Mayer’s hematoxylin (Merck, Darmstadt, Germany) for 1 min, washed in tap water, dehydrated in ethanol, cleared in xylene and coverslipped with Coverquick 2000 mounting medium (VWR International, Fontenay sous Bois, France). In every independent experiment, one representative image of a single MCA per test condition was captured using an Olympus BX50 microscope (Olympus, Tokyo, Japan) attached to a DP21 camera (Olympus, Tokyo, Japan). From those images, a quantification of the percentage of positive immunomarked cells was calculated by superimposing a grid for preventing the edging effects. A total of 200–800 cells were counted per condition (fewer cells were counted in high cytotoxicity treatments due to cell loss).

#### 4.6.6. Transmission Electron Microscopy (TEM)

MCAs were processed for TEM to assess their ultrastructural morphology. Fixation was conducted with 2.5% Glutaraldehyde in Cacodylate buffer (0.15 M, pH 7.2) for 2 h, and then the MCAs were washed twice in Cacodylatebuffer (30 min each). Post-fixation was performed with 1% osmium tetroxide (Agar Scientific, Stansted, UK) in Cacodylate buffer for 2 h. MCAs were then washed with the same buffer and dehydrated in graded ethanol series up to 100% (50% ethanol; 70% ethanol; 95% ethanol; absolute ethanol; absolute ethanol—30 min each). Until this step, all procedures were conducted at 4°C. Then, MCAs were placed at room temperature and the dehydrating agent was replaced for propylene oxide (Merck, Darmstadt, Germany) (two baths of 30 min each). Following the resin impregnation, MCAs were subjected to three mixtures of propylene oxide and epoxy resin with increasing resin concentration (propylene oxide + epon 3:1; 1:1; 1:3—1 h each mixture) and ultimately embedded in only resin (epon—1 h; epon—10 min at 60°C). After, TEM blocks were obtained by placing the MCAs in rubber molds and then tranferringing them to the oven where they stayed for 48 h at 60 °C for resin polymerization. Semithin (1.25 µm) and ultrathin (90 nm) sections were obtained with a diamond knife (Diatome, Nidau, Switzerland) on an ultramicrotome EM UC7 (Leica, Nussloch, Germany). Ultrathin sections were placed on 200 mesh hexagonal copper grids (Agar Scientific, Stansted, UK) and contrasted with 3% aqueous uranyl acetate (20 min) (Merck, Darmstadt, Germany) and Reynolds’ lead citrate (10 min) (Merck, Darmstadt, Germany). Grids were analyzed under an electron microscope JEOL 100CXII (JEOL, Tokyo, Japan), operated at 60 kV, and photographs of representative ultrastructural features were taken with the Orius SC1000 CCD digital camera (Gatan, Pleasanton, CA, USA).

### 4.7. Statistical Analysis

The descriptive and analytical statistics were performed with Past3 (version 3.19) freeware [94] and GraphPad Prism 6.0 software (GraphPad Software, La Jolla, CA, USA). The normality and homogeneity of variance were tested by the Shapiro–Wilk and the Levene tests, respectively. All the results were obtained from at least three independent experiments and expressed as mean + standard deviation (SD), except for the MCAs areas that were presented in median, maximum, minimum, and interquartile range (Q3–Q1). Significant differences (*p* < 0.05) were assessed by one-way ANOVA, followed by the post hoc Holm–Šídák multiple comparison test. In selected cases, the significance of the difference between two groups of interest was tested using the Student’s t-test together with the sequential Holm–Bonferroni correction. The latter was applied via a free spreadsheet calculator [95,96].

## Figures and Tables

**Table 1:** Tested concentrations of Fx, Cis and Dox in the combinatorial experiments.

	Compound	Fucoxanthin (Fx)	Cisplatin (Cis)	Doxorubicin (Dox)
Cell Line				
MCF7		1; 10 µM	10; 20 µM	0.01; 0.1 µM
SKBR3				
MDA-MB-231				0.1; 1 µM
MCF12A			1; 10 µM	0.01; 0.1 µM

## Data Availability

The data presented in this study are available on request from the corresponding author.

## Sample Availability

Not available.
